# The Role of Lipids, Lipid Metabolism and Ectopic Lipid Accumulation in Axon Growth, Regeneration and Repair after CNS Injury and Disease

**DOI:** 10.3390/cells10051078

**Published:** 2021-05-01

**Authors:** Debasish Roy, Andrea Tedeschi

**Affiliations:** 1 Department of Neuroscience, Wexner Medical Center, The Ohio State University, Columbus, OH 43210, USA; debasish.roy@osumc.edu; 2 Discovery Theme on Chronic Brain Injury, The Ohio State University, Columbus, OH 43210, USA

**Keywords:** lipids, axon growth and regeneration, mitochondria transport, myelin formation, adipose tissue, CNS trauma and disease

## Abstract

Axons in the adult mammalian nervous system can extend over formidable distances, up to one meter or more in humans. During development, axonal and dendritic growth requires continuous addition of new membrane. Of the three major kinds of membrane lipids, phospholipids are the most abundant in all cell membranes, including neurons. Not only immature axons, but also severed axons in the adult require large amounts of lipids for axon regeneration to occur. Lipids also serve as energy storage, signaling molecules and they contribute to tissue physiology, as demonstrated by a variety of metabolic disorders in which harmful amounts of lipids accumulate in various tissues through the body. Detrimental changes in lipid metabolism and excess accumulation of lipids contribute to a lack of axon regeneration, poor neurological outcome and complications after a variety of central nervous system (CNS) trauma including brain and spinal cord injury. Recent evidence indicates that rewiring lipid metabolism can be manipulated for therapeutic gain, as it favors conditions for axon regeneration and CNS repair. Here, we review the role of lipids, lipid metabolism and ectopic lipid accumulation in axon growth, regeneration and CNS repair. In addition, we outline molecular and pharmacological strategies to fine-tune lipid composition and energy metabolism in neurons and non-neuronal cells that can be exploited to improve neurological recovery after CNS trauma and disease.

## 1. Introduction

**Figure 1:**
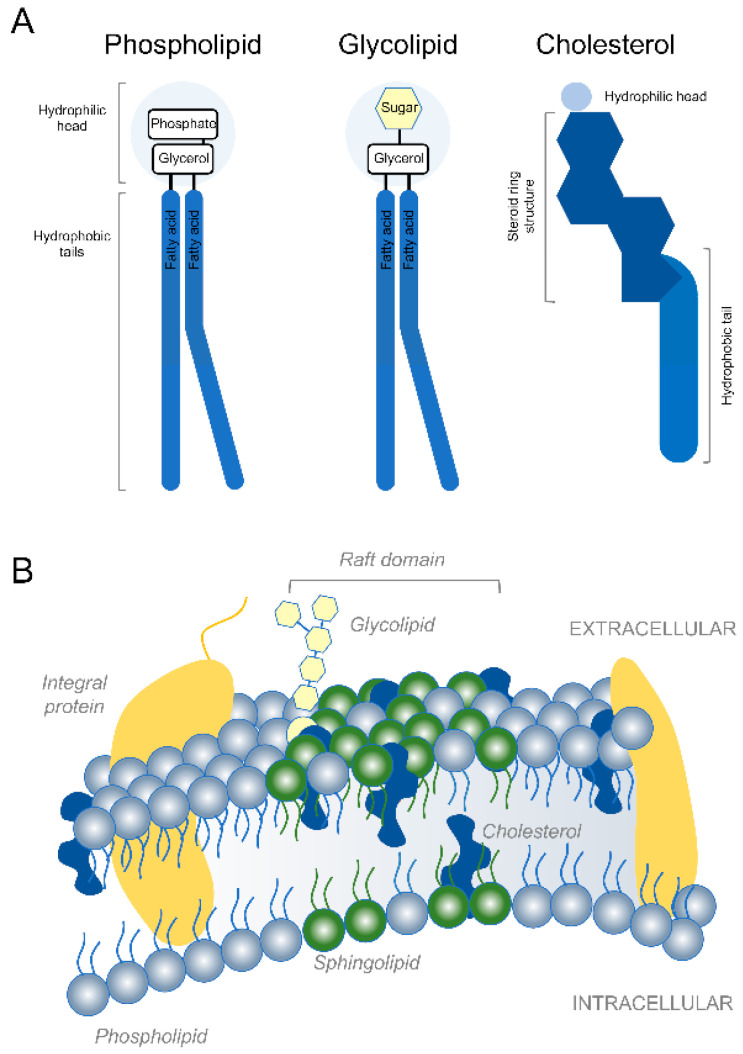
Lipids and plasma membrane. (**A**) Schematic representation of the three major kinds of membrane lipids. (**B**) Illustration of the typical lipid bilayer of the plasma membrane.

Developing axons in the mammalian nervous system can extend very long distances [1]. After reaching their targets, axons integrated into functional circuits continue to extend via mechanisms of stretch growth as the body continues to grow [2,3]. Continuous addition of new axonal membrane is necessary to fuel axon growth during development as well as long distance axon regeneration in adulthood [4,5,6]. Similarly, complex dendritic growth and myelin formation also require large amounts of membrane. Phospholipids, glycolipids and cholesterol constitute the major kinds of membrane lipids [7] (Figure 1). Phospholipids are the most abundant and are generally composed of two fatty acids that can differ in length and a phosphate group attached to a glycerol backbone [8]. Due to their shape and amphipathic nature, phospholipids spontaneously aggregate to form bilayers in aqueous environments. Made of a hydrophobic lipid tail and one or more sugar groups linked by a glycosidic bond, glycolipids are found on the outer leaflet of eukaryotic cell membranes where they confer membrane stability and facilitate cell–cell communication and signal transduction. By making the lipid bilayer less deformable and less fluid, cholesterol integration alters the permeability-barrier properties of the cellular membranes. Membrane proteins tend to accumulate within specialized microdomains of the plasma membrane called lipid rafts that are rich in cholesterol and sphingolipids. Whereas axons in the mammalian peripheral nervous system spontaneously regenerate over long distances [9,10], axons in the central nervous system (CNS) fail to mount a successful regenerative response [11,12,13]. Axon regeneration failure causes long term structural and functional impairment after a variety of CNS trauma including brain and spinal cord injury (SCI) [14,15]. Previous work has demonstrated that accumulation of certain lipids and dysregulation of lipid metabolism not only contribute to developmental disorders [16,17], axon growth and regeneration failure after CNS trauma [6,18], but also neurodegenerative diseases [19,20]. Lipids are also implicated in myelin formation and serve as bioactive molecules and energy substrates [21,22]. Here, we discuss classical and novel roles of lipids, lipid metabolism and ectopic lipid accumulation in axon growth/regeneration and CNS repair. In addition, we outline molecular and pharmacological strategies that fine tune lipid composition and metabolism in neurons and non-neuronal cells to favor neurological recovery after CNS trauma and disease.

## 2. Membrane Expansion

**Figure 2:**
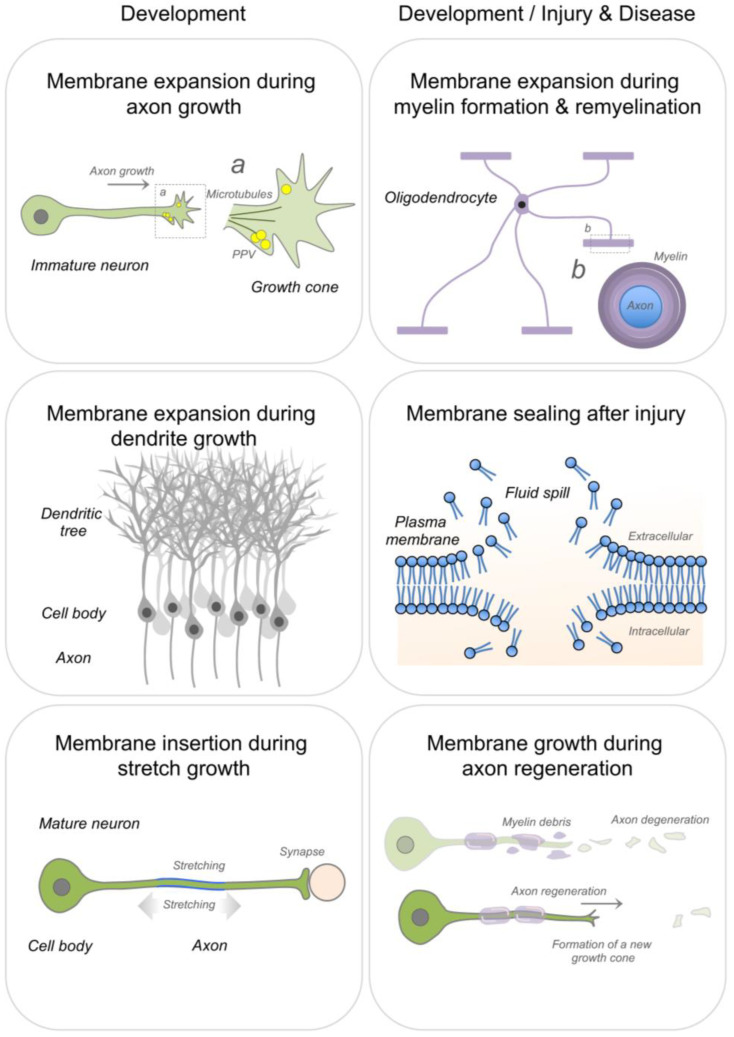
Membrane expansion during nervous system development and repair.

Continuous addition of new membrane is required during axon and dendrite growth [23,24], stretch growth of integrated axons [3] and axon regeneration [6,25,26,27] (Figure 2). Newly synthesized lipids for membrane expansion are anterogradely transported to the distal axonal compartment via lipid containing vesicles, enabling rapid elongation of growing axons [5,23,27,28,29]. In addition, axons can locally synthesize lipids, such as the major membrane phospholipid phosphatidylcholine [27]. Cholesterol can also be supplied via uptake of lipoprotein particles during rapid membrane biogenesis. Along this line, low-density lipoprotein receptors have been found to densely cluster at the tip of peripheral axons during regeneration [30]. Large amounts of lipid supply are also necessary during myelin growth and repair [31,32,33,34] (Figure 2).

A schematic representation of membrane expansion during axonal and dendritic growth, stretch growth of integrated axons, myelin formation and repair, sealing of membrane ruptures and axon regeneration. PPV: plasmalemmal precursor vesicles.

### 2.1. Axon Growth & Regeneration

**Figure 3:**
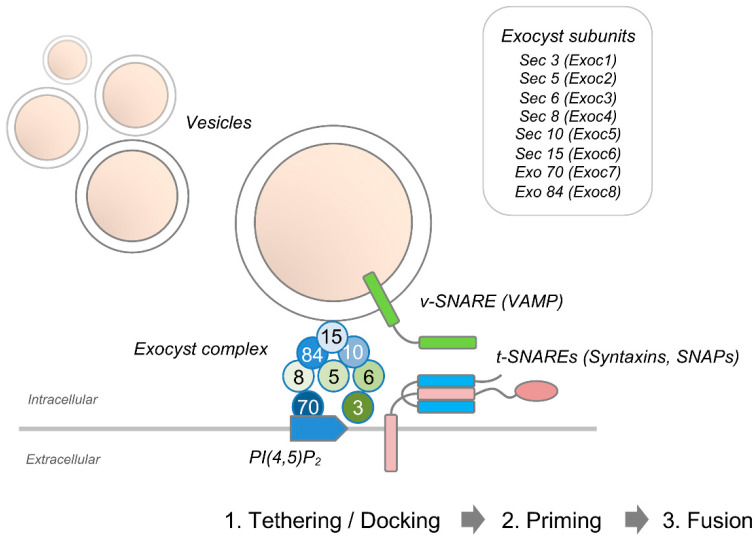
The exocyst protein complex.

Membrane expansion in developing axons is mediated by polarized exocytosis of plasmalemmal precursor vesicles that are anterogradely transported to the growth cone [35,36]. New membrane insertion may also be added at the cell body and along the neurites [37]. Specific effector complexes including the evolutionarily conserved exocyst complex that tethers post-Golgi secretory vesicles to the plasma membrane mark sites of exocytosis [38]. The octameric exocyst complex is comprised of Sec3, Sec5, Sec6, Sec8, Sec10, Sec15, Exo70, and Exo84 [39,40] (Figure 3). These units are targeted to sites of exocytosis via different mechanisms. Whereas Sec3 and Exo70 are recruited to the plasma membrane upon binding to phosphatidylinositol 4,5-bisphosphate (PI(4,5)P2) [41], the remaining exocyst components directly associate with exocytic vesicles [42,43]. Spatial and temporal regulation of the exocyst complex not only allow restricting its function to specific areas of membrane expansion such as the neuronal growth cone, but also to correctly integrating extracellular cues that cause changes in growth rate and guidance. The small cdc42-like GTPase TC10 (also known as RhoQ) controls translocation of Exoc3, Sec8, and Exo70 to the plasma membrane [44,45]. Intra-axonal synthesis of TC10 is required for exocyst function. Specifically, phosphoinositide 3-kinase (PI3K)-dependent activation of the Rheb-mTOR pathway has been shown to trigger local translation of TC10 during axon outgrowth in dorsal root ganglion (DRG) neurons [46]. Upon tethering, plasmalemmal precursor vesicles fuse with the acceptor membrane, leading to expansion of the plasma membrane. During neuronal polarization of cultured hippocampal pyramidal neurons, Dupraz et al. demonstrated that insulin-like growth factor 1-mediated activation of TC10 triggers translocation of exo70 to the plasma membrane in the distal axon and growth cone [47]. 

A schematic representation of the assembly of the exocyst octameric protein complex that controls the tethering of secretory vesicles to the plasma membrane prior to SNARE-mediated fusion. 

Soluble N-ethylmaleamide attachment proteins (SNAP), vesicle associated membrane proteins (VAMP) and syntaxis form the core machinery of vesicle fusion also known as the SNARE (soluble N-ethylmaleamide-sensitive factor attachment protein receptors) complex [48]. Whereas SNAP and syntaxin are associated with the target membrane, VAMP associates with the vesicle membrane. In yeast, mutations that block vesicle tethering prevent the assembly of the SNARE complex [49]. Both syntaxin, a membrane-integrated protein that plays an instrumental role in exocytosis [50], and Sec1, a Sec1/Munc18 family protein that binds to SNAREs and stimulates membrane fusion [51], were shown to co-immunoprecipitate with the exocyst complex [38,39,52].

After axotomy, rapid sealing of disrupted membranes is necessary to restore boundary integrity of the axonal compartment, to equilibrate ion concentration and to counteract osmotic stress [53]. These steps are required to mount a successful axon regeneration response. After small (<1 nm) membrane injuries, lipids in the bilayer spontaneously rearrange to eliminate the free edge with water during membrane resealing. In contrast, active repair mechanisms like those requiring calcium-dependent exocytosis are needed for larger injuries [54,55]. Vesicles accumulate to form multivesicular structures that fuse with the ruptured plasma membrane [56]. Hence, calcium-dependent membrane resealing involves vesicle delivery, docking, and fusion, similar to neurotransmitter exocytosis [57].

Formation of a new growth cone at the axonal stump also requires insertion of new membrane into the neurolemma [58,59]. A proteomics and lipidomics analysis of growth cone pathways during optic nerve regeneration has recently provided new insights into changes in lipid metabolic processes and lipid composition of the growth cone plasmalemma across different developmental ages [60]. 

Another recent study in adult CNS neurons has shed light on the specific changes in lipid metabolism that contribute to axon regeneration. After axotomy, expression of the phosphatidic acid phosphatase enzyme lipin 1 increase in retinal ganglion cells [6]. Mechanistically, lipin 1 catalyzes the conversion of phosphatidic acid to diglycerides [61]. Instead of producing phospholipids necessary for membrane expansion, increased lipin 1 favors the production of triglyceride storage lipids and therefore contributes to axon regeneration failure in the adult mammalian CNS [6]. Neuronal depletion of lipin 1 promotes axon regeneration after optic nerve injury in mice by regulating glycerolipid metabolism and, more specifically, triglyceride hydrolysis and phospholipid synthesis [6]. This suggests that triglycerides may provide lipid precursors for phospholipid synthesis that is necessary for membrane biosynthesis during axon regeneration. 

During early stages of development, immature neurons exhibit an extraordinary axon growth and regenerative capacity [15,62,63]. As neurons mature, however, a development dependent decline in the expression of genes controlling axon growth is, at least in part, responsible for axon regeneration failure in the adult [64,65]. In turn, the identification of positive regulators and molecular mechanisms controlling axon growth and regeneration has been the subject of intensive investigation [12,25,66,67,68,69,70]. In exploring the mechanisms that control intrinsic axon growth, a recent study has discovered that the stemness-associated gene *Prom1,* which encodes the membrane glycoprotein Prominin-1, acts as a positive regulator of peripheral nerve regeneration in adulthood [71]. *Prom1* forced expression enhances axon regeneration via Smad2-dependent signaling and inhibits the expression of genes related to cholesterol biosynthesis [71]. 

In the adult CNS, the vast majority of cholesterol is present in myelin sheaths and plasma membranes. After SCI, disruption of cellular membranes and myelin sheaths cause an excess of cholesterol to accumulate in the extracellular space. Of interest, cholesterol depletion has been shown to promote axon growth in vitro and regeneration of peripheral nerves in vivo [72]. By lowering cholesterol synthesis, molecular and pharmacological strategies have proven effective in promoting survival and CNS regeneration of retinal ganglion cells after optic nerve injury [73]. Work done in mice deficient for CNS myelin and myelin basic protein (also known as shiverer mice [74]) has demonstrated the inhibitory action of CNS myelin lipids, in particular cholesterol and sphingomyelin, to axon regeneration after SCI [75]. Shiverer mice, but also mice administered 2-hydroxypropyl-β-cyclodextrin to scavenge lipids at the injury site, showed increased regeneration of dorsal column sensory axons after SCI [75].

The neuronal cell bodies in sensory, sympathetic and parasympathetic ganglia are wrapped by satellite glial cells [76]. The importance of satellite glia in nervous system function and dysfunction has attracted attention lately [77]. A recent study has demonstrated that fatty acid synthesis in satellite glial cells contributes to axon elongation during regeneration of peripheral nerves [25]. Hence, satellite glia may facilitate peripheral nerve regeneration by paracrine lipid transfer via lipoprotein particles to DRG neurons.

Together, experimental evidence suggests that rewiring lipid metabolism can be manipulated for therapeutic gain as it favors axon regeneration both in the central and peripheral nervous systems. Whether pharmacological and molecular strategies lowering cholesterol synthesis promote neurological recovery after SCI is unknown and deserves further attention in future investigations. 

### 2.2. Dendritic Growth

The development of complex dendritic branches for sampling and processing synaptic inputs also requires a large lipid supply. Recent studies in *Drosophila* have shown that dendrite expansion in larva neurons relies on cell-autonomous production of lipids [24,78]. Specifically, dendrite formation in dendritic arborization neurons requires sterol regulatory element binding protein (SREBP), as shown by the presence of dendrites lacking complex morphology in neurons from *srebp* mutant. SREBP acts as a basic helix–loop–helix leucine zipper transcription factor that binds to sterol regulatory elements to control the expression of genes that encode key enzymes of both fatty acid and cholesterol metabolism [79,80,81]. Interestingly, structural changes in dendrite morphology are accompanied with hypersensitivity to noxious stimuli in *srebp* mutant larvae. In turn, cell-autonomous production of fatty acids is necessary for correct dendritic development and function. A genetic screen in dendritic arborization sensory neurons of the *Drosophila* larval peripheral nervous system has identified the *easily shocked* (*eas*) gene, which encodes the ethanolamine kinase (also known as the first enzyme of the Kennedy pathway involved in the de novo biosynthesis of the phospholipid phosphotidylethanolamine [82]), as a central regulator of dendritic morphogenesis [78]. Total phosphotidylethanolamine is decreased in *eas* mutants [83], causing SREBP to translocate to the nucleus where it activates the expression of lipogenic genes [84]. *Eas* mutant flies experience seizures and show defects in dendrite morphogenesis characterized by a decrease in the number of branches and total dendrite length in dendritic arborization sensory neurons [78]. Defects in these mutants are associated with increased lipogenesis with altered membrane phospholipid composition and increased calcium influx [78]. Forcing SREBP transcriptional activity by expressing a constitutively active form of SREBP leads to a decrease in the number of neurite branches and total length [78], further suggesting SREBP-mediated transcription is crucial during dendrite morphogenesis [85,86]. Thus, accumulating evidence suggests that lipid production must be fine-tuned during development as lipid levels that are too low or too high can lead to structural and functional impairment in dendritic structures.

### 2.3. Myelin Formation and Repair

Myelin is constituted by multiple overlapping layers of a specialized membrane spiraling around axons [87]. Myelin not only increases axonal resistance, but also enables high density sodium channel clustering at nodes of Ranvier, thereby enabling high speed propagation of electrical signals [88,89,90,91]. CNS myelination is crucial for metabolic support and key for functional recovery after injury and disease [92,93,94,95]. Myelin basic protein and proteolipid proteins represent the two major CNS myelin-specific proteins [96,97,98]. In contrast, all major classes of lipids are present in myelin membranes, suggesting myelin does not contain specific lipids. When compared to other membranes, however, the lipid:protein ratio is much higher in myelin membranes, with lipids contributing 70–80% of the dry weight of myelin [8,99,100]. Myelin membranes contain high levels of cholesterol (>25% by weight) and glycosphingolipids, specifically galactosylceramide and ether-glycerophospholipids, whose deficiency impairs myelin formation [101,102,103]. Cholesterol and lipid accumulation accounts for myelin membrane expansion during CNS development [31,32,33,34]. Large amounts of cholesterol may also contribute to myelin membrane curvature and fluidity [104,105]. Once incorporated into the myelin membrane, cholesterol turnover is very slow [106]. Transcriptional and posttranscriptional mechanisms controlling sterol homeostasis have been identified in eukaryotes [107]. There may be potential mechanisms to remove free cholesterol from oligodendrocytes via secretion of cholesterol-rich exosomes into the extracellular milieu [108]. 

During normal brain development, astrocyte-derived fatty acids constitute a significant portion of lipids incorporated into CNS myelin [109]. Remarkably, oligodendrocytes are capable of bypassing inhibition of lipid synthesis in astrocytes by incorporating circulating lipids into myelin membranes [109]. This suggests that personalized lipid-enriched diets may rescue, at least in part, myelination defects. Nevertheless, accumulation of ceramides, the precursor of sphingolipids, and its direct product sphingosine in the spinal cord of a rat experimental model of demyelinating disorders causes apoptosis of oligodendrocytes [110]. 

As result of CNS trauma and disease, myelin sheaths along the axon can be severely damaged. De novo fatty acid synthesis via fatty acid synthase is critical for CNS myelin formation after demyelination injury. Genetic depletion of fatty acid synthase from OPC has no effect on proliferation and differentiation along the oligodendrocyte lineage, yet is necessary for accurate CNS myelination [111]. Treatment with cholesterol lowering drugs such as statins that target HMG-CoA reductase [112], the rate-limiting enzyme of cholesterol biosynthesis, negatively impacts myelin formation and repair after SCI [113,114,115]. 

In mammals, the ability to regenerate and repair the CNS declines with age [116,117]. In old mice, large quantities of cholesterol-rich myelin debris hijack phagocyte-mediated clearance [118]. Under such pathological conditions, free cholesterol starts to form crystals that cause lysosomal rupture with consequent inflammasome stimulation [118]. In this context, cholesterol lowering drugs used to treat hypercholesterolemia may be repurposed for regenerative medicine applications. 

Recent evidence suggests that a delicate balance in lipid synthesis and homeostasis is crucial during myelin formation and remyelination during CNS development, as well as after injury or disease. Strategies targeting lipid composition of the myelin membrane may exert contradictory responses where a lipid-enriched diet may rescue myelination defects while excess lipids trigger oligodendrocyte death and inflammation. Learning how to fine tune lipid composition in myelin membranes via extracellular lipid supply, lowering cholesterol levels or promoting its clearance with spatial precision without causing adverse effects represent important areas of future investigation. 

## 3. Energy Metabolism and Mitochondrial Transport

As discussed above, lipids are necessary for proper sealing of the plasma membrane, formation of a new growth cone and regeneration after axonal injury. These energy demanding processes require optimal mitochondrial transport and adenosine triphosphate (ATP) production [58,119,120,121,122,123]. During glucose metabolism, most ATP is produced by oxidative phosphorylation in mitochondria. Under pathological conditions, however, high levels of fatty acids impair several processes involved in oxidative phosphorylation during ATP synthesis. Accumulated fatty acids depolarize mitochondrial membranes and bind to electron transport chain complexes to generate peroxide and hydroxyl radicals [124,125]. Both neurons and astrocytes slowly convert fatty acids into energy substrates [126]. Fatty acid oxidation, however, cannot guarantee rapid ATP generation during fast and sustained neuronal firing [124]. In response to neuronal activity, astrocytes utilize fatty acids stored in lipid droplets via mitochondrial β-oxidation and activate a detoxification program to protect neurons from fatty acid-mediated toxicity during sustained neuron firing [127]. Using experimental models of amyotrophic lateral sclerosis, recent studies in rats have showed that excess accumulation of lipid droplets, particularly cholesteryl esters linked to polyunsaturated fatty acids, in spinal cord astrocytes is associated with cellular stress and inflammation [128,129]. Therefore, elevated fatty acid levels plus their use as an energy substrate following CNS trauma and disease not only impair neuronal function, but also promote oxidative stress and neurodegeneration. 

As axons reach their target field during late stages of development, formation of collateral branches and synaptic contacts allow neurons to establish complex connectivity patterns [130,131,132]. The liver kinase B1 (LKB1) links cellular metabolism and energy homeostasis to cell polarity and growth [133,134,135,136]. The LKB1-NUAK1 kinase pathway promotes cortical axon branching by inducing mitochondria immobilization, including at nascent presynaptic sites [137]. LKB1 phosphorylates AMP-activated protein kinase [138] that acts as a crucial regulator of axonal regenerative signaling [139] and lipid and glucose metabolism in all eukaryotic cells [140]. LKB1 forced expression in adult corticospinal neurons promotes regeneration of corticospinal axons after murine SCI [141]. Regeneration of serotonergic and tyrosine hydroxylase-positive axons is also achieved in mice with systemic overexpression of LKB1 [141]. By inhibiting ATP-consuming biosynthetic pathways, AMP-activated protein kinase restores ATP levels via simultaneous activation of signaling pathways that regenerate ATP from the breakdown of macromolecules [140]. It is also important to note that AMP-activated protein kinase can also activate transcription factors that act as master regulators of metabolism [142,143]. 

Mitochondria trafficking and anchoring along the axons needs to be tightly regulated to respond to altered energy requirements [144]. To meet energy and calcium buffering needs, mitochondria movement through the dense axoplasm cannot rely on passive diffusion rates. Instead, ATP-hydrolyzing motor proteins of the kinesin-1 family control anterograde transport of mitochondria from the soma to the distal axon [145,146]. In addition, dynein motors control retrograde transport back to the soma [147,148]. The mitochondrial Rho GTPase 1 (Miro 1) associates with milton (TRAK1/2) and the motor proteins kinesin and dynein to form the mitochondria motor/adaptor complex [149]. Axonal microtubules are necessary for long distance fast axonal transport of mitochondria [150]. 

Mitochondrial positioning along the axon is important as it enables local energy supply [151]. Accumulating evidence indicates that proper mitochondrial transport and high energy supply are necessary to fuel axon growth and regeneration. When actively growing, chick sympathetic neurons preferentially localize mitochondria toward the growth cone [152]. Analysis of mitochondria behavior in these axons indicates that mitochondrial movement is balanced so that net transport is anterograde in growing axons [152]. After axonal injury, acute depolarization of mitochondria causes an energy crisis along injured axons [123]. Depolarized mitochondria not only fail to produce adequate levels of ATP, but also contribute to axonal degeneration by spilling byproducts of mitochondrial metabolism such as toxic reactive oxygen species [153]. Overexpression of Miro 1 or silencing of the mitochondria-anchoring protein syntaphilin enhances mitochondria transport and restores energy balance, thereby promoting axon regrowth [123]. Additional evidence indicates that boosting mitochondrial transport effectively promotes neuron survival and axon regeneration after axotomy both in worms and mice [119,122]. 

How does cellular metabolism contribute to neuron repair after axotomy? By linking glucose metabolism to the hexosamine biosynthetic pathway, *O*-linked β-*N*-acetylglucosamine (*O*-GlcNAc) post-translational modification of serine and threonine residues of proteins acts as a nutrient sensor and metabolic mediator [154]. One day after laser axotomy in *worms*, a decrease in O-GlcNAc levels promotes axon regeneration by adopting glycolysis as the primary source of energy [154]. Whilst mutant worms with decreased *O*-GlcNAc levels fail to regenerate after blocking glucose transport or inhibiting glycolysis, increasing *O*-GlcNAc levels act on mitochondrial function and enhance axon regeneration in *Caenorhabditis elegans* through FOXO/DAF-16–dependent mechanisms [154]. The fact that *O*-GlcNAc levels drive distinct branches of the insulin pathway to promote regeneration in worms may help explain these seemingly contradictory results. 

As glial cells are metabolically coupled to axons, they may provide energy metabolites to support axon survival and function [94,155,156,157]. In fact, glia metabolic functions can be reprogrammed in favor of glycolysis to promote anatomical and functional regeneration after CNS injury in both flies and mice [158].

Together, the above examples underscore the importance of achieving optimal mitochondrial transport and glial metabolism as they are necessary for energy production to fuel axon growth and regeneration. Whether mitochondrial transport may be linked to nutrient and energy sensing is not known. It is also not clear whether mitochondria have memories, allowing them to respond rapidly to changes in environmental nutrient and energy levels. The extent to which boosting energy metabolism and mitochondria trafficking may be sufficient to promote functionally relevant regeneration and CNS repair under different experimental conditions awaits confirmation. 

## 4. Energy Balance, Lipid Accumulation and Adipose Tissue

After CNS trauma and disease, altered lipid metabolism and lipid accumulation in neurons and various tissues in the body can be harmful, as it causes permanent cellular damage [159]. To understand the molecular mechanisms that cause or contribute to lipid accumulation, one must understand the concept of energy balance and, in particular, energy intake and expenditure. When the energy intake exceeds the physiological demand, excess energy is stored as fat [160]. Energy can be expended via increasing basal metabolism or physical activity (for example, thermogenesis) [161]. Basal metabolism and thermogenesis refer to the metabolic processes involved in the maintenance of body functions and energy production in response to cold or food intake, respectively. To maintain a healthy energy balance, food intake and energy expenditure have to be correctly regulated through the body. Detrimental changes in either of these can alter the energy balance, thereby causing adipose tissue deposition and increased fatty acid release into the circulation.

### 4.1. Lipolysis, Adaptive Thermogenesis and Innervation of Adipose Tissue

**Figure 4:**
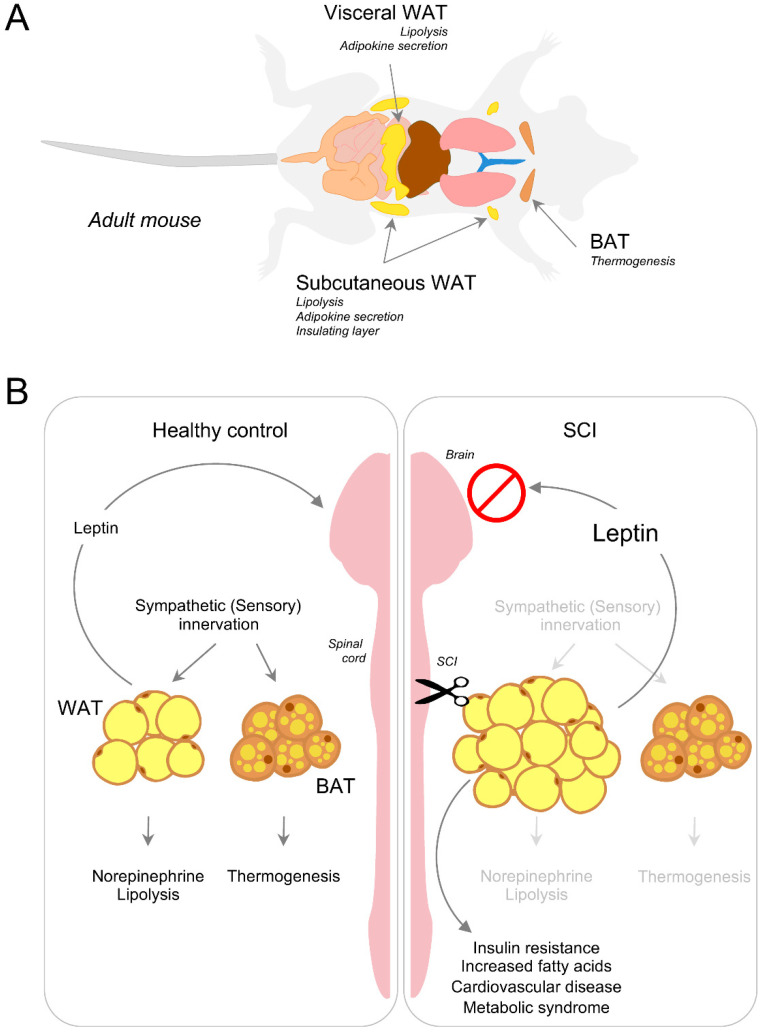
Adipose tissue depots, energy balance and leptin sensing. (**A**) Representation of adipose depots located under the skin and within the abdomen in adult mice. Whereas WAT can be subdivided into subcutaneous, bone marrow and visceral depots, BAT mainly localizes in the interscapular region. (**B**) Under normal physiological conditions, sympathetic innervation regulates WAT lipolysis and BAT thermogenesis. The contribution of sensory innervation is far less understood. Leptin action on the neurons located in the hypothalamus is critical for homeostatic regulation of energy balance. After SCI, interruption of adipose tissue innervation causes a breakdown in energy homeostasis leading to adipose tissue accumulation and ectopic lipid spillover to vital organs. If protracted, such detrimental conditions contribute to the development of insulin resistance, increased fatty acids, cardiovascular complications and metabolic syndrome.

Under normal physiological conditions, excess energy is stored in depots of white adipose tissue (WAT) in the form of triacylglycerides. WAT depots include abdominal and subcutaneous fat [162,163] (Figure 4). To mobilize stored energy during exercise or fasting, WAT releases fatty acids via lipolysis, a biochemical pathway through which triacylglycerol stored in lipid droplets is hydrolyzed into a glycerol and three fatty acids for usage by other organs [164,165]. While it is present in all tissues and cell types, lipolysis occurs mainly in the adipose tissue [166]. Non-esterified fatty acids are not only used as energy substrates but also as precursors for membrane synthesis. The inter-organ demand and utilization of fat breakdown products are therefore necessary for survival. When, however, the energy balance shifts to excess lipid storage, the demand-driven supply chain breaks down leading to ectopic assimilation of lipids in non-adipose organs altering the whole-body energy balance.

Generation of body heat by external stimuli like temperature or food is called adaptive thermogenesis and is further classified as either shivering or non-shivering thermogenesis. By controlling the non-shivering thermogenic function, brown adipose tissue (BAT) plays a crucial role in maintaining whole-body energy balance. BAT’s unique ability to produce heat relies on the mitochondrial uncoupling protein 1 (UCP1), which disengages oxidative phosphorylation from the electron transport chain to produce heat instead of energy in the form of ATP [167]. Increase in BAT volume is associated with increased lipolysis, free fatty acid cycling and oxidation, as well as adipose tissue insulin sensitivity [168].

Recent evidence suggests that innervation of adipose tissue may play a critical role in lipid mobilization and endocrine signaling [169,170,171]. After CNS trauma and neurodegenerative disease, disruption of adipose tissue innervation can lead to deleterious consequences including insulin resistance and cardiovascular disease [172,173] (Figure 4). Despite having a ‘normal’ body mass index, SCI individuals often develop severe adiposity. Given that the majority of these individuals has moderate to normal feeding behavior without hyperphagia [174], the energy imbalance owing to the rise in energy intake cannot explain the substantial increase in adiposity. Reduced physical activity may contribute to this phenomenon. However, adiposity in age-matched healthy individuals with the same activity profile is considerably lower when compared to SCI individuals. Pioneering work by Bartness et al. demonstrated that Siberian hamsters experience seasonal variation in adiposity and that such variation can be reproduced in the laboratory by altering the photoperiod from long to short days [175]. The authors tested photoperiod inducible melatonin and its circulating intermediaries (for example, glucocorticoids and thyroid hormone) as ‘effector’ molecules that can drive the intra-abdominal fat mobilization in hamsters. Surprisingly, experimental evidence has shown that these molecules do not account for the observed changes [175], suggesting other mechanisms may be responsible for adipose mobilization. 

More than 100 years ago, Dogiel reported neurons of unknown origin innervate WAT [176]. Decades later, Corell et al. corroborated this observation by demonstrating that electrical stimulation of WAT causes the release of fat breakdown products, suggesting a neuronal mechanism may be controlling lipolysis [177]. Building on this seminal contribution, numerous studies have demonstrated that sympathetic nerve endings connect in the WAT parenchyma and that WAT innervation is required for lipolysis [178,179,180]. Using retrograde tracing techniques, postganglionic sympathetic neurons were found to innervate WAT. The majority of the neurons innervating WAT originate from thoracic (T)12–lumbar (L)1 spinal ganglia [181,182]. New viral and genetic tools allow precise localization of preganglionic and postganglionic neurons innervating adipose tissue. Transneuronal tracing using pseudorabies virus injected in epididymal WAT has demonstrated that sympathetic innervation of WAT connects to several regions in the brain such as the hypothalamus, which controls feeding behavior to maintain energy balance and homeostasis [183]. Disruption of this system could contribute to adiposity after injury and disease. 

Sensory neurons also innervate WAT. In fact, T13-L3 neuronal cell bodies can be visualized upon retrograde tracing in WAT [184]. Similarly, herpes simplex viral injection in the inguinal fat pad has revealed that WAT efferent neurons connect with DRG neurons in the lower thoracic dorsal horn and upper lumbar spinal cord [185]. Interestingly, the sensory circuitry innervating WAT is sensitive to leptin, an adipose tissue hormone that sends feeding signals to the hypothalamus [186,187] (Figure 4). While leptin injection in WAT induces c-Fos expression in DRG neurons, leptin intraperitoneal injection fails to elicit such a response, thereby suggesting a functional role of sensory innervation in WAT [188].

Unlike WAT, BAT segments bilaterally [189]. As norepinephrine released by the sympathetic fibers innervating BAT activates mitochondrial UCP1, denervation of the ipsilateral side of BAT lowered UCP1, norepinephrine and the overall BAT mass compared to the non-innervated contralateral side [190]; norepinephrine treatment reverses the loss of BAT activity. Sympathetic projections that innervate BAT arise from the four anterior nerve bundles of the intercostal muscles [190]. A recent study showed that sympathetic post-ganglionic neurons that innervate BAT connect the stellate ganglia along with T1-T5 spinal ganglia [169,191]. Similar to WATs, sympathetic neurons that innervate brown fat connect several key hypothalamic centers [192]. Taken together, this shows that adipose tissue connects with the CNS and the spinal cord via the sympathetic nervous system. Disruption of innervation due to CNS trauma may therefore alter systemic energy balance.

### 4.2. Disruption of Adipose Tissue Innervation and Energy Metabolism after Injury

Lipolysis and adaptive thermogenesis are necessary to control a healthy energy balance. Neurons that connect the hypothalamic regions and innervate WAT and BAT form a ‘leptin sensitive’ closed loop. The arcuate nucleus in the hypothalamus is one such CNS region that critically senses leptin levels [193] to promote sympathetic innervation of adipose tissue [170]. The ability to sense leptin, as well as leptin’s function in adipose tissue, can be compromised after CNS trauma. In turn, interruption of adipose innervation negatively impacts both areas of energy balance [194]. A rise of leptin in the serum of SCI individuals positively correlates with an increase in adiposity following SCI [195,196,197,198]. Leptin promotes UCP1 expression in BAT to trigger BAT-dependent thermogenesis. Despite having high basal levels of leptin, SCI individuals have a reduced energy expenditure profile [199]. Two possible explanations may help understand this phenomenon. First, local leptin activity on brown fat requires sympathetic innervation of BAT which is reduced after SCI. Second, BAT may become insensitive to leptin and no longer be able to respond to it. Work done in obese mice has shown that central versus peripheral administration of leptin promotes sympathetic outflow to BAT and promotes thermogenesis [200], suggesting that leptin’s action in the brain supports a positive energy balance. In turn, impaired sympathetic function can compromise leptin action on BAT to lower thermogenesis after SCI, causing a reduction in energy expenditure (Figure 4). Whether leptin exerts a direct effect on BAT sympathetic innervation is currently unknown. WAT lipolysis strongly depends on leptin action as well. Along this line, treating rat fat pads ex-vivo with leptin leads to an increase in the release of fatty acids in a dose- and time-dependent manner [201]. A recent study demonstrated that leptin’s lipolytic role is dependent on sympathetic innervation of WAT [202]. Furthermore, intraperitoneal leptin administration reverses defective metabolism in ob/ob mice (e.g., leptin deficient mice) by promoting sympathetic innervation of WAT [170]. Severed adipose innervation following a CNS trauma therefore deregulates leptin sensing, causing adipose accumulation and an overall increase in circulating fatty acids (Figure 4). Future work should aim at understanding how CNS trauma and neurodegenerative diseases impair ‘leptin sensitive’ circuits. Progress along this line may help in the development of therapeutic strategies aimed at lowering the risk of adipose spillover in visceral organs, preventing organ failure. Normalization of the energy metabolism can lower excess fatty acid buildup in the brain and spinal cord and lower the risk of neuronal comorbidities associated with CNS trauma and disease. 

### 4.3. Adiposity and CNS Injury

Individuals that sustained an injury to the brain or spinal cord may have lower life expectancy due to chronic secondary complications that develop months and years after injury. Bladder and bowel dysfunction can cause anxiety, social isolation and depression after SCI [203]. Sympathetic hypoactivity leads to low systemic arterial pressure [204,205]. Moreover, disconnection of the excitatory drive from the brainstem leads to impaired autonomic functions and low sympathetic tone [206]. Sympathetic innervation of the adipose tissue is crucial to maintain the balance in energy storage and expenditure. Loss of sympathetic innervation promotes adiposity and contributes to devastating chronic complications like insulin resistance and cardiac dysfunction. Notably, type 2 diabetes (for example, insulin resistance) and cardiac arrest are the leading cause of death in SCI individuals [207]. For simplicity, we will refer to ‘adiposity’ as the excess lipid accumulation within adipose tissue and ectopic deposition in non-adipose organs. 

It is important to note that adiposity in SCI individuals and healthy individuals is not exactly the same. Within a year after injury, SCI individuals lose bodyweight, mostly in lean muscle mass [208]. The drastic loss of muscle mass destabilizes the typical energy expenditure profile and contributes to adipose tissue accumulation in ectopic depots. Of note, the overall increase in adiposity does not affect the body mass index due to the low muscle mass in SCI individuals [209]. The body mass index cutoff range used to determine obesity in non-SCI individuals underestimates adiposity in SCI individuals [210], and therefore needs to be adjusted [211,212]. 

At least half of individuals with SCI has >30% of their total body mass as adipose tissue, indicating SCI individuals are at high risk of developing metabolic syndrome [213] (Figure 4). Adipose tissue preferentially accumulates in specific locations within the body. Using imaging methods like MRI and dual-energy X-ray absorptiometry to map regional adiposity in SCI individuals has confirmed an increase of ~50% in subcutaneous and visceral adipose tissue [213,214,215]. SCI individuals not only experience increased adiposity in these two common regions of the body, but also a rise in bone marrow adipose tissue [197]. Recent studies on obesity have demonstrated that a rise in bone marrow adiposity promotes osteoporosis and impairs several immune-associated functions [216,217]. An increase in bone marrow adiposity can have an impact on bone marrow stem cell lineage differentiation towards myelopoiesis. As such, newly differentiated myeloid cells can infiltrate into various tissues and promote pro-inflammatory conditions that further exacerbate metabolic syndrome [218,219,220]. Individuals with motor complete SCI develop 2 to 3 times more bone marrow adiposity than age-matched controls [221] and therefore, are at risk of systemic inflammation induced metabolic syndrome. Accumulating evidence suggests that a rise in adiposity in non-adipose tissues including the liver, heart and skeletal muscle is caused by neurological deficits related to SCI [209] and consequently, is independent of physical activity [222]. For example, cervical and midthoracic SCI in rats leads to excess lipid accumulation in the liver, augmented proinflammatory gene expression and increased CD68 positive macrophage infiltration [223]; these are hallmarks of chronic liver injury similar to those of fatty liver disease.

### 4.4. Ectopic Liver Fat Accumulation after SCI

A rise in triglycerides in the liver may be caused by increased storage after de novo synthesis, reduced breakdown, or impaired secretion in the form of very-low-density lipoproteins (VLDL). Under pathological conditions like obesity, fatty acids are converted into triglycerides. These are stored as neutral lipid droplets as they no longer get converted into energy substrates by oxidation. With more incoming fatty acids adding to the pool of stored lipid droplets in the liver, the de novo synthesis of lipids increases, thereby causing lipid accumulation within the liver [224,225]. As the supply of fat exceeds the demand for energy homeostasis, the liver releases lipids in a controlled manner in the form of VLDL. Several factors regulate this process, but insulin-mediated secretion of VLDL is by far the most crucial [226]. When insulin is high, VLDL secretion from the liver increases. During fasting, insulin is lowered and VLDL secretion halts to (i) prevent detrimental accumulation of triglycerides in circulation and (ii) to maintain steady energy homeostasis in the liver. 

Proper insulin signaling in the liver orchestrates the critical steps of VLDL secretion [226]. However, a pathological rise in adiposity promotes hyperinsulinemia and insulin resistance causing a detrimental increase in VLDL in the circulation. Interestingly, SCI individuals with paraplegia and complete sensorimotor injuries (for example, loss of sensation or motor function below the injury) have high serum VLDL levels [227]. With age, high de novo lipogenesis and lipid-laden liver progress towards non-alcoholic fatty liver disease. A clinical study has found that 50% of SCI individuals experience liver adiposity induced non-alcoholic fatty liver disease ~1 year after injury [228]. Accumulation of lipids in the liver at chronic stages after SCI has been reported in rodents and humans [223,228], indicating animal models are extremely valuable as they effectively reproduce mechanisms of injury and disease found in humans [229]. 

More than half of the triglycerides deposited in the liver originates from circulating fatty acids and ~25% of the triglycerides comes from de novo lipogenesis [230]. Given that excess lipids in the liver depend on circulating fatty acids derived from adipose tissue, a rise in adiposity may precede a breakdown in liver homeostasis. The inflammatory response that develops acutely after SCI exacerbates this condition [231,232]. As systemic inflammation persists well beyond the acute phase of SCI [231], liver adiposity may convert to a more severe pathology known as non-alcoholic steatohepatitis. Increased adiposity followed by a rise in leptin level activates T cells [233,234] and polarizes T cell differentiation towards a T helper cell type 1 phenotype that produces pro-inflammatory cytokines like TNF-alpha and IFNγ [228,235]. When coupled with systemic inflammation at chronic stages of SCI, liver adiposity may lead to tissue fibrosis and non-alcoholic steatohepatitis if left untreated [196,228]. 

A gradual rise in whole-body adiposity compromises the balance between fatty acid uptake and secretion with negative impacts on the liver metabolic profile. Despite recent progress, a thorough mechanistic understanding of liver pathology after SCI is still lacking. As the liver represents the primary site for drug metabolism, reducing hepatic metabolic dysfunction may boost efficacy of SCI medications and repair strategies. 

### 4.5. Glucose Intolerance, Insulin Resistance and Cardiovascular Complications

As circulating free fatty acid levels increase due to adiposity, the glucose-fatty acid cycle, also known as the ‘Randle’ cycle [236], destabilizes. A rise in plasma-free fatty acids blocks glucose oxidation and impairs glucose uptake in the liver and skeletal muscle [237,238]. The low glucose utilization in muscle and liver increases glucose intolerance and insulin resistance, a chronic condition that develops in individuals with SCI [239]. Individuals with chronic SCI display low plasma glucose clearance [240] with significantly high insulin levels [239]. Both adiposity and muscle wasting could contribute to insulin resistance. In addition, adiposity leads to cardiac lipotoxicity induced damage after SCI. 

Cardiovascular complications represent the leading cause of death in chronic SCI individuals [241,242,243]. Partial central deafferentation of spinal neurons causes atrophy of the spinal sympathetic pre-ganglionic neurons [206]. Because these neurons innervate the heart, their severe atrophy may lead to cardiac arrest. However, case report studies in humans suggest cardiac arrest at chronic stages is not due to atrophy of sympathetic pre-ganglionic neurons, as these neurons can regain normal function [206]. With a reduced sympathetic tone and low lipolysis, adipose tissue accrues lipids [202]. SCI individuals with paraplegia have elevated LDL-cholesterol and serum triglyceride levels [244]. Due to increased expression of the fatty acid transporter protein CD36, the cardiac tissue readily takes up low-density lipoproteins and fatty acids [245,246]. With increased fatty acid load, mitochondrial energy metabolism reverts to an embryonic metabolic phenotype that produces ATP by utilizing glucose [247]. Lowering β-oxidation and increasing glycolysis changes the expression of key metabolic enzymes that maintain a proper glycolytic state. Impaired β-oxidation and lipotoxicity increase myocardial triglyceride content and augment the risk of cardiac failure [247,248]. Whether these mechanisms are the primary cause for the development of cardiovascular dysfunction after SCI remains to be evaluated.

## 5. Conclusions

We have discussed evidence suggesting that reprogramming lipid metabolism, boosting mitochondrial transport and neuron-glia metabolic coupling promote survival and regeneration of injured axons. Whereas large amounts of lipids are necessary during development to grow axons, dendrites and myelin sheaths, dysregulation of lipid metabolism, impaired mitochondrial functions and excess lipid accumulation through the body including in non-adipose organs can be harmful as they cause lipotoxicity and increased levels of fatty acids. Not only do such detrimental conditions contribute to axon regeneration failure, but also to insulin resistance, cardiovascular disease and metabolic syndrome, thus leading to poor neurological outcomes and a diminished quality of life. In addition, spatial and temporal alteration of circulating fatty acids may compromise CNS function and repair under pathological conditions including CNS trauma and neurodegenerative diseases. Correct positioning of organelles for membrane trafficking, lipid exchange and energy production requires a complex interplay between various organelles and molecular motors [249]. The fabrication of new inducible and reversible systems may allow us to gain spatiotemporal control over this process [250], enabling CNS repair with precision. Moreover, a thorough mechanistic understanding of adipose tissue remodeling following CNS trauma and disease is lacking. With the development and optimization of non-invasive three-dimensional imaging of glucose uptake and neuronal tracing strategies, we will begin to understand the importance of adipose tissue innervation, in particular sensory innervation, endocrine function and inter-organ lipid homeostasis. Regaining metabolic control and energy balance may be necessary to promote neuron repair and full body recovery after CNS trauma and disease.
